# Preventive effect of rebamipide on NSAID-induced lower gastrointestinal tract injury using FAERS and JADER

**DOI:** 10.1038/s41598-022-06611-y

**Published:** 2022-02-16

**Authors:** Toru Imai, Katsuyuki Hazama, Yasuhiro Kosuge, Shinichiro Suzuki, Susumu Ootsuka

**Affiliations:** 1 Department of Pharmacy, Nihon University Itabashi Hospital, Itabashi-ku, 173-8610 Tokyo Japan; 2 Laboratory of Pharmacology, School of Pharmacy, Nihon University, Funabashi-shi, 274-8555 Chiba Japan

**Keywords:** Translational research, Small intestine

## Abstract

Non-steroidal anti-inflammatory drugs (NSAIDs) are widely used for their antipyretic, analgesic, and anti-inflammatory properties. However, various aspects of NSAID-induced lower gastrointestinal tract injury remain unclear, and effective prophylaxis has not been established. Based on its pharmacological effect and clinical trials, rebamipide may prevent lower gastrointestinal tract injury, although this evidence is limited by the small scale of trials. The present study used the FDA Adverse Event Reporting System (FAERS) and the Japanese Adverse Event Reporting Database (JADER) to assess the efficacy of rebamipide in combination with loxoprofen and diclofenac in preventing NSAID-induced lower gastrointestinal tract injury. The calculated reporting odds ratio and 95% confidence interval (CI) for rebamipide in combination with loxoprofen and diclofenac were 1.15 (95% CI 0.88–1.51) and 1.28 (95% CI 0.82–2.01) for FAERS, and 0.50 (95% CI 0.35–0.71) and 0.43 (95% CI 0.27–0.67) for JADER, respectively. No signal was detected when combining drugs. These results suggest a prophylactic effect of rebamipide on NSAID-induced lower gastrointestinal tract injury.

## Introduction

Non-steroidal anti-inflammatory drugs (NSAIDs) are widely applied for their antipyretic, analgesic, and anti-inflammatory properties^1^. They have been known to cause upper gastrointestinal tract injury; however, advances in capsule endoscopy have revealed that NSAIDs can also elicit lower gastrointestinal tract injury^2,3^. The latter has been linked to fewer mucosal protective factors caused by decreased prostaglandin production, oxidative stress due to mitochondrial dysfunction, and an abundance of gram-negative bacteria following disruption of intestinal microflora homeostasis^4–6^. Nevertheless, several aspects of NSAID-induced lower gastrointestinal tract injury remain unclear, and an effective prophylaxis has not been established.

Rebamipide has antioxidant effects by protecting mucous membranes through increased production of prostaglandins and by inhibiting the production of superoxide as a free radical scavenger^7–9^. In this way, rebamipide counteracts some of the causes of NSAID-induced lower gastrointestinal tract injury. Rebamipide has been shown to be effective in preventing lower gastrointestinal tract injury when used in combination with diclofenac, a common NSAID^10–12^. Such pharmacological evidence and the findings from clinical trials suggest that rebamipide may have a beneficial protective function; however, this evidence is limited by the small size and unreliability of trial reports. There are various approaches for analyzing prophylaxis against adverse drug reactions. Although randomized controlled trials (RCTs) provide the best evidence, it is sometimes difficult from an ethical perspective to conduct large-scale clinical studies on prophylaxis^13,14^. Moreover, it is not possible to comprehensively analyze all types of patients, as children, pregnant women, and individuals with underlying conditions are generally excluded from clinical trials^13,14^.

In recent years, large real-world adverse drug reaction databases, such as the U.S. Food and Drug Administration (FDA) Adverse Event Reporting System (FAERS) and the Japanese Adverse Drug Event Report (JADER) of the Pharmaceuticals and Medical Devices Agency (PMDA), have come to include an increasing amount of data^15–18^. Spontaneous adverse event reports include data on patients normally excluded from clinical trials, making them more likely to reflect clinical practice. Such databases are now regarded as a suitable alternative to RCTs for preventive drug discovery^19–22^. FAERS is the world's largest database of spontaneous adverse event reports, with 13 million cases currently registered. Comparatively, JADER has fewer entries, but it is useful for drugs, such as rebamipide, which are frequently prescribed in Japan. Although there are some differences in the nature of data published by FAERS and JADER, they can serve as complementary sources of information^23^.

In the present study, we employed these two databases of adverse drug reactions to analyze NSAID-induced lower gastrointestinal tract injury in relation to concomitant use of rebamipide and evaluated the possible protective effect of this agent.

## Results

**Figure 1:**
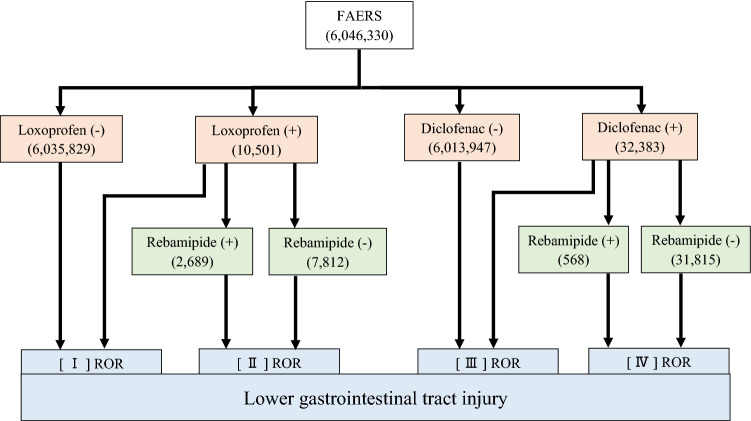
Flow chart summarizing the determination of RORs for lower gastrointestinal tract injury categorized according to drug usage in FAERS. Numbers in parentheses indicate the number of adverse drug reactions reported. [I] ROR shows the odds ratio of lower gastrointestinal tract injury for loxoprofen alone and for drugs excluding loxoprofen. [II] ROR shows the odds ratios for loxoprofen with and without rebamipide. [III] ROR shows the odds ratio of lower gastrointestinal tract injury for diclofenac alone and for drugs excluding diclofenac. [IV] ROR shows the odds ratios for diclofenac with and without rebamipide.

The flow chart illustrating the use of the FAERS database to analyze the reporting odds ratio (ROR) of NSAID-induced lower gastrointestinal tract injury is shown in Fig. 1. The ROR (95% confidence interval [CI]) for loxoprofen and diclofenac was 4.23 (95% CI 3.75–4.78) and 4.70 (95% CI 4.40–5.02), respectively. The lower limit of the 95% CI of ROR for both drugs was > 1 and, hence, the adverse event signal was detected (Table 1, [I] [III]). Assessing the combined effect of rebamipide and loxoprofen or diclofenac on NSAID-induced lower gastrointestinal tract injury yielded a ROR (95% CI) of 1.15 (95% CI 0.88–1.51) and 1.28 (95% CI 0.82–2.01). The lower limit of the 95% CI was < 1, and no signal was detected (Table 1, [II] [IV]).

**Table 1:** Number of cases and ROR (95% CI) of each NSAID-induced lower gastrointestinal tract injury adverse event reported in FAERS.

	LGTI	Other AE	LGTI (%)	ROR	95% CI
**[I]**					
Loxoprofen (+)	269	10,232	2.56	4.23	3.75–4.78
Loxoprofen (−)	37,288	5,998,541	0.68		
**[III]**					
Diclofenac (+)	907	31,476	2.80	4.70	4.40–5.02
Diclofenac (−)	36,650	5,977,297	0.61		
**[II]**					
Loxoprofen					
Rebamipide (+)	76	2613	2.83	1.15	0.88–1.51
Rebamipide (−)	192	7620	2.46		
**[IV]**					
Diclofenac					
Rebamipide (+)	20	548	3.52	1.28	0.82–2.01
Rebamipide (−)	880	30,935	2.76		

Lower gastrointestinal tract injury of single-drug NSAIDs [I], [III]. Lower gastrointestinal tract injury with concomitant use of rebamipide and NSAIDs [II], [IV].

*LGTI* lower gastrointestinal tract injury, *AE* adverse event.

**Figure 2:**
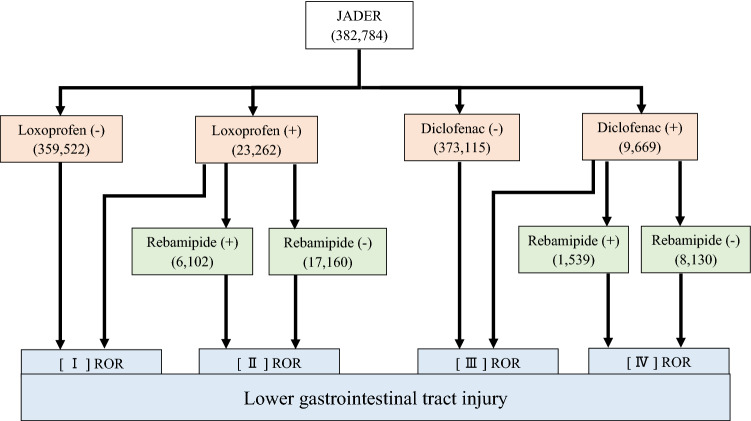
Flow chart summarizing the determination of RORs for lower gastrointestinal tract injury categorized according to drug usage in JADER. Numbers in parentheses indicate the number of adverse drug reactions reported. [I] ROR shows the odds ratio of lower gastrointestinal tract injury for loxoprofen alone and for drugs excluding loxoprofen. [II] ROR shows the odds ratios for loxoprofen with and without rebamipide. [III] ROR shows the odds ratio of lower gastrointestinal tract injury for diclofenac alone and for drugs excluding diclofenac. [IV] ROR shows the odds ratios for diclofenac with and without rebamipide.

The same analysis was performed using the JADER database and the corresponding flow chart is shown in Fig. 2. The ROR (95% CI) for loxoprofen and diclofenac was 1.61 (95% CI 1.40–1.84) and 4.84 (95% CI 4.26–5.50), respectively. The lower limit of the 95% CI for both loxoprofen and diclofenac was > 1, and the signal was detected (Table 2, [I] [III]).

**Table 2:** Number of cases and ROR (95% CI) of each NSAID-induced lower gastrointestinal tract injury adverse event reported in JADER.

	LGTI	Other AE	LGTI (%)	ROR	95% CI
**[I]**					
Loxoprofen (+)	236	23,026	1.02	1.61	1.40–1.84
Loxoprofen (−)	2271	357,251	0.63		
**[III]**					
Diclofenac (+)	274	9395	2.91	4.84	4.26–5.50
Diclofenac (−)	2233	370,882	0.60		
**[II]**					
Loxoprofen					
Rebamipide (+)	36	6066	0.59	0.50	0.35–0.71
Rebamipide (−)	200	16,960	1.17		
**[IV]**					
Diclofenac					
Rebamipide (+)	21	1518	1.38	0.43	0.27–0.67
Rebamipide (−)	253	7877	3.21		

Lower gastrointestinal tract injury of single-drug NSAIDs [I], [III]. Lower gastrointestinal tract injury with concomitant use of rebamipide and NSAIDs [II], [IV].

*LGTI* lower gastrointestinal tract injury, *AE* adverse event.

The ROR (95% CI) for lower gastrointestinal tract injury with loxoprofen and diclofenac in combination with rebamipide was 0.50 (95% CI 0.35–0.71) and 0.43 (95% CI 0.27–0.67). The lower limit of the 95% CI was < 1, and no signal was detected (Table 2, [II] [IV]).

## Discussion

Reports about the clinical significance and frequency of adverse events with NSAIDs resulting in lower gastrointestinal tract injury are increasing^24^, and they present a higher mortality rate than upper gastrointestinal events, suggesting the need for early prophylaxis^25^. At present, there is no prophylaxis for NSAID-induced lower gastrointestinal tract injury because acid secretion inhibitors are not expected to be effective. In this study, we analyzed the preventive effect of the combination of NSAIDs and rebamipide on NSAID-induced lower gastrointestinal tract injury using the FAERS and JADER databases. The results showed that the risk of lower gastrointestinal tract injury was reduced when rebamipide was combined with loxoprofen and diclofenac, confirming a potential preventive effect.

NSAID-induced lower gastrointestinal tract injury has been reported in 40‒53% of patients taking diclofenac for 2 weeks^3,26^, and in 71% of patients taking multiple NSAIDs for more than 3 months^27^. These values indicate that NSAIDs cause frequent lower gastrointestinal tract injuries, regardless of treatment duration. In the present study, the RORs of loxoprofen and diclofenac were consistent with the findings from previous reports, and an adverse event signal was obtained with both drugs. Hence, this study can be considered a good reflection of actual clinical practice.

In recent years, data mining using large-scale databases has been attracting attention as an exploratory study method for the prevention of adverse drug reactions^21,22^. Zhao et al. investigated the preventive effect of exenatide combination on the side effects of rosiglitazone, such as myocardial infarction and stroke, using FAERS^21^. The results showed that the combination of rosiglitazone and exenatide eliminated the signals of myocardial infarction and stroke. Furthermore, validation using a mouse model proved that exenatide inhibits the increase in blood coagulation caused by rosiglitazone and has a preventive effect on myocardial infarction and stroke. Nagashima et al. also conducted an analysis using FAERS to search for prophylactic drugs that can reduce the risk of hyperglycemia caused by atypical antipsychotics^22^. The results showed that the diabetes-related signal of quetiapine disappeared when combined with vitamin D. Furthermore, the results of validation using a mouse model established that vitamin D improves insulin resistance to quetiapine. As shown in these reports, the results of basic research correlated with the disappearance of side effect signals when certain drugs were used in combination, so database analysis may play a complementary role. Pharmacological evidence suggests a protective effect of rebamipide against NSAID-induced lower gastrointestinal tract injury^28,29^. Clinical trials have also shown the prophylactic effects of rebamipide, but they were carried out only in Japan, and the number of cases was reduced^10–12^. To date, no large-scale clinical trials have been conducted. In this study, the analysis was conducted using FAERS and JADER with reference to basic research and small clinical trials of rebamipide. The results suggest that rebamipide may be effective as a prophylactic agent according to FAERS and JADER data, as the combination of NSAIDs and rebamipide abolished the signals associated with lower gastrointestinal tract injury (Tables 1, 2).

Data from FAERS and JADER may differ with respect to race, social background, and medical circumstances^30^. Analyzing interstitial pneumonia using FAERS and JADER, Matsui et al. found very different rates of gefitinib-induced injury (7.4% and 45.6%, respectively)^31^, indicating strong regional variations between the two databases. In JADER, most reports are from Japan, whereas in FAERS, 70% are from the U.S.A. Rebamipide was developed in Japan and was launched there in 1990, while in other countries it entered the market only after the year 2000, explaining the limited number of cases in FAERS (Figs. 1 and 2). FAERS entries on adverse reaction associated with rebamipide are insufficient; however, the incidence can be estimated more accurately if analytical results from JADER are also taken into account^15^. In fact, complementing information from one database with the other will provide a better understanding of the discrepancy in adverse events between Japan and other countries, and thus ensure a more targeted approach in clinical practice^16,31^. Supporting the use of both databases and confirming the reliability of this study, it should be noted that the present results are similar in spite of different patient backgrounds. Thus, pharmaco-epidemiological studies using databases, such as JADER and FAERS, offer excellent tools for detecting trends on adverse drug reactions and preventive medications. However, the present study has some limitations that need to be considered. It should be kept in mind that these data are derived from an observational study, not an analysis using RCTs, and the possibility of bias in reporting rates should be recognized. In addition, because the adverse event spontaneous reporting database is voluntary, it is not possible to capture all adverse events that occur. Furthermore, the lack of comprehensive medical records and medication histories limits the scope of the analysis, as the dosage and duration of use of NSAIDs and rebamipide are unknown.

Nevertheless, FAERS and JADER databases represent a rich and invaluable post-market resource for drug safety research. The huge number of cases obtained by using these databases is solid information and has been reported to be useful^19–22^. In this study, the combined use of NSAIDs and rebamipide eliminated the signals associated with lower gastrointestinal tract injury. This was consistent with the results of previous clinical studies, and we believe that it contains important findings. We believe that these findings can stimulate future research on rebamipide, ultimately helping to prevent or control serious complications, such as ulceration, bleeding, or perforation, caused by NSAID-induced lower gastrointestinal tract injury.

## Methods

### Data acquisition and preprocessing

FAERS data were downloaded from the FDA website (http://www.fda.gov/). The FAERS database contains data tables named 'DEMO', 'DRUG', 'REAC', 'OUTC', 'RPSR', 'THER', and 'INDI'. In this study, we used the DEMO, DRUG, and REAC files. The DEMO file contains basic patient information such as age, date of adverse event, and country of adverse event; the DRUG file contains the name of the drug, route of administration, and dose; and the REAC file contains the name of the adverse event. Microsoft Office Access 2016 was used to create the FAERS dataset used in this study. In the case of duplicate reports from the same patient, only the most recent adverse event was analyzed, in line with FDA recommendations. The JADER database was downloaded from the PMDA website (https://www.pmda.go.jp/index.html). It consists of four files: 'DEMO', 'DRUG', 'REAC', and 'HIST'; and data tables are divided by ID number. We included reports labeled as "suspicious drug" in the drug involvement section based on Hosomi et al.^32^. Multiple reports that were registered in the database in duplicate were deleted based on case information, and each case was regarded as one report.

### Database search

All reports added to the FAERS and JADER databases between April 1, 2004 and March 31, 2016 were accessed and downloaded. The adverse event entries used for extraction were selected from the 39 terms corresponding to "lower gastrointestinal tract injury" in the Preferred Term (PT) of the ICH International Medical Dictionary for Regulatory Activities (MedDRA ver. 22.1): Rectal haemorrhage (PT 10038063), Anal haemorrhage (PT 10049555), Lower gastrointestinal haemorrhage (PT 10050953), Large intestinal haemorrhage (PT 10052534), Small intestinal haemorrhage (PT 10052535), Intestinal haemorrhage (PT 10059175), Mesenteric haemorrhage (PT 10060717), Anal ulcer (PT 10002180), Anorectal ulcer (PT 10002582), Rectal perforation (PT 10038073), Rectal ulcer (PT 10038080), Rectal ulcer haemorrhage (PT 10038081), Anal ulcer haemorrhage (PT 10063896) Anal erosion (PT 10067272), Duodenal perforation (PT 10013832), Duodenal ulcer (PT 10013836), Duodenal ulcer haemorrhage (PT 10013839), Duodenal ulcer perforation (PT 10013849), Erosive duodenitis (PT 10062532), Ulcerative duodenitis (PT 10080994), Ileal perforation (PT 10021305), Ileal ulcer (PT 10021309), Ileal ulcer perforation (PT 10021310), Intestinal perforation (PT 10022694), Intestinal ulcer (PT 10022714), Jejunal perforation (PT 10023174), Jejunal ulcer (PT 10023177), Jejunal ulcer perforation (PT 10023178), Large intestinal ulcer (PT 10023799), Large intestine perforation (PT 10023804), Small intestinal perforation (PT 10041103), Small intestine ulcer (PT 10041133), Large intestinal ulcer perforation (PT 10052497), Small intestinal ulcer perforation (PT 10052498), Intestinal ulcer perforation (PT 10061248), Large intestinal ulcer haemorrhage (PT 10061262), Small intestinal ulcer haemorrhage (PT 10061550), Large intestine erosion (PT 10076369), and Lower gastrointestinal perforation (PT 10078414).

### Statistical analyses

The ROR is a widely used method in adverse drug event signal detection. It has been employed extensively in studies based on FAERS and JADER data^15–18^. When the lower limit of the 95% CI for the adjusted ROR was > 1, the adverse event was considered to be significantly more reported following use of the drug of interest compared to all other drugs. The ROR score is defined as $\frac{A/B}{C/D}$, where ‘‘A’’ is the number of safety reports in patients who received rebamipide and manifested the NSAID-induced lower gastrointestinal tract injury adverse event; ‘‘B’’ is the number of safety reports, in which patients received rebamipide but did not present the NSAID-induced lower gastrointestinal tract injury adverse event; ‘‘C’’ is the number of safety reports, in which patients did not receive rebamipide and had the NSAID-induced lower gastrointestinal tract injury event; and ‘‘D’’ is the number of safety reports, in which patients did not receive rebamipide and did not present the NSAID-induced lower gastrointestinal tract injury adverse event.

## Data Availability

The datasets generated and analyzed during the current study are available from the corresponding author on reasonable request.
